# Tris(ethane-1,2-di­amine-κ^2^ *N*,*N*′)zinc(II) tetra­chlorido­zincate(II)

**DOI:** 10.1107/S2414314620006185

**Published:** 2020-05-12

**Authors:** Y. AaminaNaaz, K. Rajkumar, S. Thirumurugan, K. Anbalagan, A. SubbiahPandi

**Affiliations:** a Department of Physics, Presidency College (Autonomous), Chennai 600 005, India; b Department of Chemistry, Pondicherry University, Pondicherry 605 014, India; Goethe-Universität Frankfurt, Germany

**Keywords:** crystal structure, ethane-1,2-diamine, zinc(II) complex, hydrogen bonding, N—H⋯Cl and C—H⋯Cl inter­actions

## Abstract

The title complex, [Zn(C_2_H_8_N_2_)_3_][ZnCl_4_], exists as discrete ions. The [Zn(C_2_H_8_N_2_)_3_]^2+^ cation exhibits a distorted octa­hedral shape. In the [ZnCl_4_]^2−^ anion, the Zn^II^ atom is in an almost regular tetra­hedral environment. The crystal packing is consolidated by N—H⋯Cl and C—H⋯Cl hydrogen bonds.

## Abstract 2

The crystal structure of the title compound is consolidated by N—H⋯Cl and C—H⋯Cl hydrogen bonding inter­actions.

## Graphical abstract

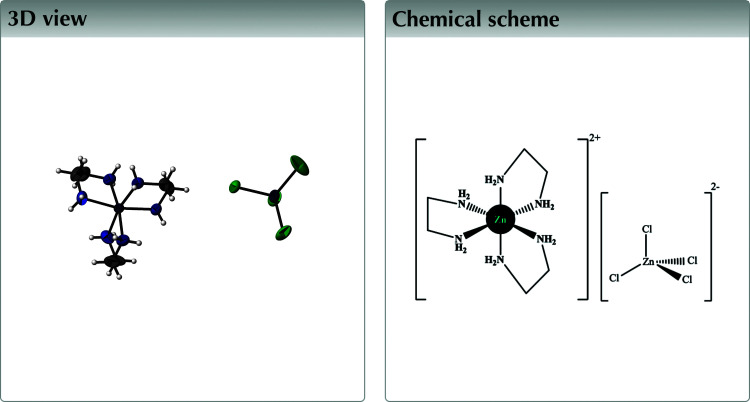

## Structure description

Ethyl­enedi­amine (en) is a common chelating ligand that is widely used in transition-metal complexes. It cannot only chelate metal cations by two nitro­gen atoms, but also offers hydrogen atoms to form N—H⋯*X* hydrogen bonds. Metal complexes containing an ethyl­enedi­amine (–NCH_2_CH_2_N) backbone have attracted significant inter­est as potential anti­cancer agents because of their rich redox chemistry and relative ease of manipulation (Mihajlović *et al.*, 2012 ▸; Beaumont *et al.*, 1976 ▸). Metal-containing compounds offer many advantages over conventional carbon-based compounds, their ability to coordinate ligands in a three-dimensional configuration allowing the functionalization of groups that can be tailored to defined mol­ecular targets (Fricker, 2007 ▸; Meggers, 2009 ▸).

Metals such as zinc act as a key structural component in many proteins and enzymes, including transcription factors, cellular signalling proteins and DNA repair enzymes (Prasad, 1995 ▸; Prasad & Kucuk, 2002 ▸). Zinc deficiency during pregnancy may produce serious defects and foetal loss (Hernick & Fierke, 2005 ▸). Zinc also possesses anti­viral, anti­bacterial and wound-healing properties with zinc complexes also being used in the treatment of gastrointestinal disorders, acne and infertility (Cunnane, 1988 ▸). Against this background, the X-ray structural characterization of the title compound has been carried out in order to determine the mol­ecular conformation, binding modes and hydrogen-bonding inter­actions.

**Figure 1:**
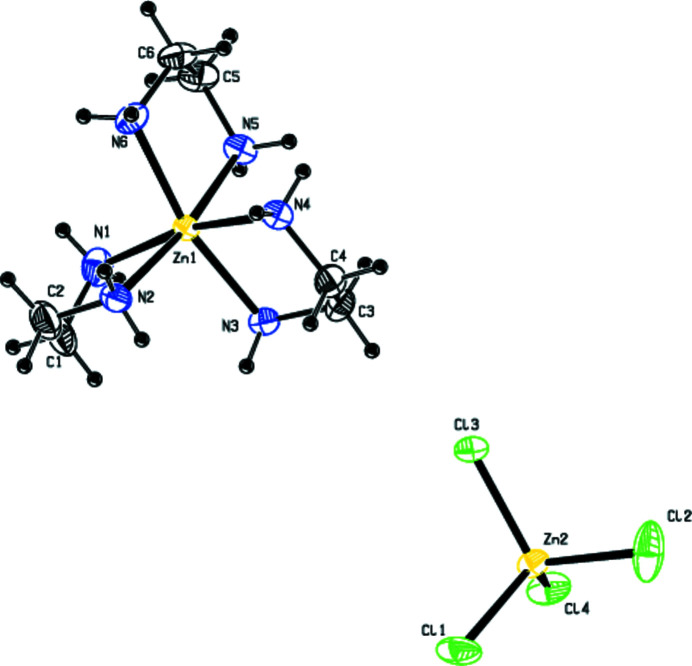
View of the mol­ecular structure of the title complex, showing the atom labelling. Displacement ellipsoids are drawn at the 30% probability level.

Fig. 1 ▸ shows the mol­ecular entities of the title complex, [Zn(C_2_H_8_N_2_)_3_][ZnCl_4_], which comprises an ZnCl_4_^2−^ anion and a [Zn(en)_3_]^2+^complex cation. The Zn^II^ atom of the tetra­chlorido­zincate(II) anion is in an almost regular Cl_4_ tetra­hedral environment, with Zn—Cl bond lengths in the range 2.255 (1)-2.272 (9) Å. The zinc cation displays a distorted octa­hedral coordination geometry defined by six N atoms from three ethyl­enedi­amine ligands, with Zn—N distances in the range of 2.173 (3)–2.219 (3) Å. The N—Zn—N angles of the en ligands are about 80°. They are noticeably smaller than the ideal octa­hedral angle of 90°. The five-membered chelate rings are non-planar, with N—C—C—N torsion angles of −57.5 (4), −55.4 (4) and −55.9 (5)°. All of the three en ligands assume a synclinal conformation about the C—C bond.

**Figure 2:**
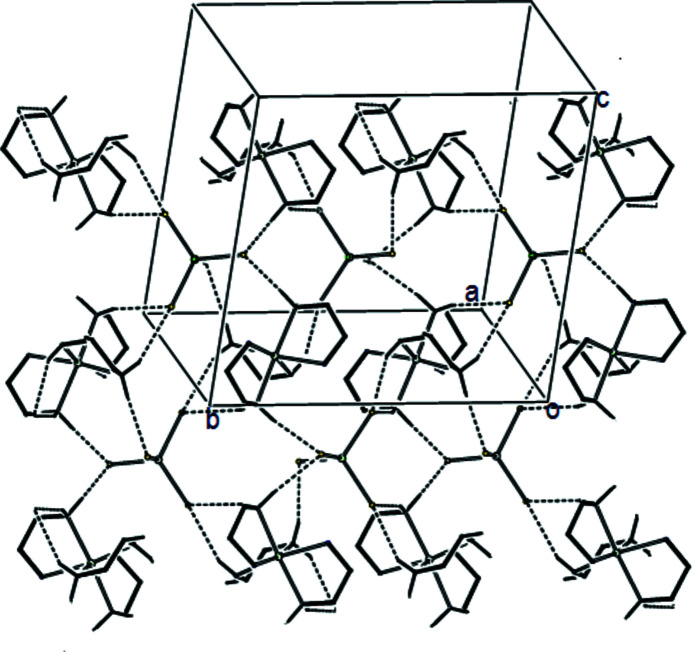
The crystal packing of title complex, viewed approximately down the *a* axis, with hydrogen bonds (Table 1 ▸) shown as dashed lines.

In the crystal structure, adjacent ions are connected *via* inter­molecular hydrogen bonds. The N—H⋯Cl hydrogen-bonding inter­actions between the N atoms of the ethyl­enedi­amine ligands and Cl atoms of the tetra­chlorido­zincate anion connect the mol­ecules, together with the weak C—H⋯Cl intra­molecular inter­actions, generating a three-dimensional network (Fig. 2 ▸, Table 1 ▸).

## Synthesis and crystallization

Zinc chloride (1.36 g, 2 mol) was dissolved in 25 ml of EtOH/H_2_O (1:4 *v*/*v*) mixture. To this solution, ethyl­enedi­amine (1.0 ml, 3 mol) in 25 ml of an HCl/EtOH (2:3 *v*/*v*) mixture was added dropwise. The mixture was stirred and heated to 338 K for 2 h and allowed to stand at room temperature until colourless crystals separated (3–4 weeks). Crystals suitable for single-crystal XRD were collected after recrystallization using acidified water.

## Refinement

Crystal data, data collection and structure refinement details are summarized in Table 2 ▸.

## Supplementary Material

Crystal structure: contains datablock(s) I, global. DOI: 10.1107/S2414314620006185/bt4091sup1.cif

Structure factors: contains datablock(s) I. DOI: 10.1107/S2414314620006185/bt4091Isup2.hkl

CCDC reference: 2001547

Additional supporting information:  crystallographic information; 3D view; checkCIF report

## Figures and Tables

**Table 1:** Hydrogen-bond geometry (Å, °)

*D*—H⋯*A*	*D*—H	H⋯*A*	*D*⋯*A*	*D*—H⋯*A*
N1—H1*D*⋯Cl1^i^	0.89	2.88	3.617 (3)	142
N1—H1*D*⋯Cl3^i^	0.89	2.95	3.677 (3)	140
N2—H2*C*⋯Cl1^ii^	0.89	2.56	3.426 (3)	165
N2—H2*D*⋯Cl3^iii^	0.89	2.51	3.395 (3)	171
N3—H3*C*⋯Cl1^ii^	0.89	2.67	3.500 (3)	155
N3—H3*D*⋯Cl1^i^	0.89	2.99	3.670 (3)	135
N3—H3*D*⋯Cl2^i^	0.89	2.85	3.600 (3)	143
N4—H4*C*⋯Cl4^iv^	0.89	2.63	3.437 (3)	152
N4—H4*D*⋯Cl2^iii^	0.89	2.76	3.581 (3)	153
N5—H5*C*⋯Cl4^iv^	0.89	2.58	3.431 (3)	160
N5—H5*D*⋯Cl3^i^	0.89	2.50	3.360 (3)	162
N6—H6*C*⋯Cl2^v^	0.89	2.91	3.646 (3)	141
N6—H6*D*⋯Cl3^iii^	0.89	2.91	3.652 (3)	142
N6—H6*D*⋯Cl4^iii^	0.89	2.90	3.609 (3)	138
C3—H3*B*⋯Cl3	0.97	2.82	3.662 (3)	146

Symmetry codes: (i) 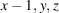 ; (ii) 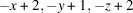 ; (iii) 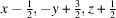 ; (iv) 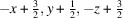 ; (v) 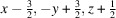 .

**Table 2:** Experimental details

Crystal data	
Chemical formula	[Zn(C_2_H_8_N_2_)_3_][ZnCl_4_]
*M*_r_	452.85
Crystal system, space group	Monoclinic, *P*2_1_/*n*
Temperature (K)	293
*a*, *b*, *c* (Å)	8.6916 (4), 14.6035 (9), 14.0382 (7)
β (°)	91.201 (4)
*V* (Å^3^)	1781.45 (16)
*Z*	4
Radiation type	Mo *K*α
μ (mm^−1^)	3.29
Crystal size (mm)	0.20 × 0.12 × 0.10

Data collection	
Diffractometer	Oxford Diffraction Xcalibur diffractometer with EOS detector
Absorption correction	Multi-scan (*CrysAlis PRO*; Oxford Diffraction, 2009 ▸)
*T*_min_, *T*_max_	0.629, 0.720
No. of measured, independent and observed [*I* > 2σ(*I*)] reflections	10159, 3119, 2582
*R*_int_	0.027
(sin θ/λ)_max_ (Å^−1^)	0.595

Refinement	
*R*[*F* ^2^ > 2σ(*F* ^2^)], *wR*(*F* ^2^), *S*	0.031, 0.075, 1.04
No. of reflections	3119
No. of parameters	163
H-atom treatment	H-atom parameters constrained
Δρ_max_, Δρ_min_ (e Å^−3^)	0.65, −0.35

Computer programs: *CrysAlis CCD* and *CrysAlis RED* (Oxford Diffraction, 2009 ▸), *SHELXS97* (Sheldrick, 2008 ▸), *SHELXL2014/7* (Sheldrick, 2015 ▸) and *PLATON* (Spek, 2020 ▸).

## full crystallographic data

### Crystal data

**Table:**

[Zn(C_2_H_8_N_2_)_3_][ZnCl_4_]	*F*(000) = 920
*M**_r_* = 452.85	*D*_x_ = 1.688 Mg m^−^^3^
Monoclinic, *P*2_1_/*n*	Mo *K*α radiation, λ = 0.71073 Å
*a* = 8.6916 (4) Å	Cell parameters from 2582 reflections
*b* = 14.6035 (9) Å	θ = 3.9–25.0°
*c* = 14.0382 (7) Å	µ = 3.29 mm^−^^1^
β = 91.201 (4)°	*T* = 293 K
*V* = 1781.45 (16) Å^3^	Block, colourless
*Z* = 4	0.20 × 0.12 × 0.10 mm

### Data collection

**Table:**

Oxford Diffraction Xcalibur diffractometer with EOS detector	2582 reflections with *I* > 2σ(*I*)
Radiation source: fine-focus sealed tube	*R*_int_ = 0.027
ω and φ scans	θ_max_ = 25.0°, θ_min_ = 3.9°
Absorption correction: multi-scan (CrysAlis Pro; Oxford Diffraction, 2009)	*h* = −10→9
*T*_min_ = 0.629, *T*_max_ = 0.720	*k* = −16→17
10159 measured reflections	*l* = −16→16
3119 independent reflections	

### Refinement

**Table:**

Refinement on *F*^2^	0 restraints
Least-squares matrix: full	Hydrogen site location: inferred from neighbouring sites
*R*[*F*^2^ > 2σ(*F*^2^)] = 0.031	H-atom parameters constrained
*wR*(*F*^2^) = 0.075	*w* = 1/[σ^2^(*F*_o_^2^) + (0.0348*P*)^2^ + 0.9424*P*] where *P* = (*F*_o_^2^ + 2*F*_c_^2^)/3
*S* = 1.04	(Δ/σ)_max_ = 0.001
3119 reflections	Δρ_max_ = 0.65 e Å^−^^3^
163 parameters	Δρ_min_ = −0.35 e Å^−^^3^

### Special details

**Table:**

Geometry. All esds (except the esd in the dihedral angle between two l.s. planes) are estimated using the full covariance matrix. The cell esds are taken into account individually in the estimation of esds in distances, angles and torsion angles; correlations between esds in cell parameters are only used when they are defined by crystal symmetry. An approximate (isotropic) treatment of cell esds is used for estimating esds involving l.s. planes.
Refinement. All H atoms were fixed geometrically and allow to ride on their parent C and N atoms, with C—H distances of 0.97 Å and N—H distances of 0.90 Å, and with *U*_iso_(H)= 1.2 *U*_eq_(parent atom).

### Fractional atomic coordinates and isotropic or equivalent isotropic displacement parameters (Å^2^)

**Table:**

	*x*	*y*	*z*	*U*_iso_*/*U*_eq_	
C1	0.2695 (6)	0.5672 (3)	1.1390 (3)	0.0762 (13)	
H1A	0.1936	0.5263	1.1654	0.091*	
H1B	0.3421	0.5307	1.1037	0.091*	
C2	0.3510 (5)	0.6156 (3)	1.2166 (3)	0.0711 (12)	
H2A	0.2773	0.6473	1.2556	0.085*	
H2B	0.4059	0.5717	1.2565	0.085*	
C3	0.6033 (4)	0.7154 (3)	0.9023 (2)	0.0504 (9)	
H3A	0.5510	0.7528	0.8546	0.061*	
H3B	0.6785	0.6777	0.8706	0.061*	
C4	0.6819 (4)	0.7751 (3)	0.9736 (3)	0.0515 (9)	
H4A	0.7429	0.7377	1.0173	0.062*	
H4B	0.7508	0.8166	0.9416	0.062*	
C5	0.1177 (4)	0.8682 (3)	0.9718 (3)	0.0620 (11)	
H5A	0.0297	0.8334	0.9930	0.074*	
H5B	0.0824	0.9108	0.9231	0.074*	
C6	0.1881 (4)	0.9199 (3)	1.0543 (3)	0.0588 (10)	
H6A	0.2718	0.9578	1.0323	0.071*	
H6B	0.1115	0.9596	1.0820	0.071*	
N1	0.1919 (4)	0.6337 (2)	1.0733 (2)	0.0579 (8)	
H1C	0.1079	0.6565	1.0997	0.069*	
H1D	0.1649	0.6066	1.0187	0.069*	
N2	0.4594 (4)	0.68128 (19)	1.17780 (17)	0.0474 (7)	
H2C	0.5472	0.6531	1.1646	0.057*	
H2D	0.4797	0.7247	1.2207	0.057*	
N3	0.4909 (3)	0.65660 (18)	0.95024 (18)	0.0420 (6)	
H3C	0.5393	0.6120	0.9820	0.050*	
H3D	0.4269	0.6313	0.9075	0.050*	
N4	0.5683 (3)	0.82833 (18)	1.02757 (18)	0.0399 (6)	
H4C	0.5424	0.8788	0.9955	0.048*	
H4D	0.6088	0.8449	1.0837	0.048*	
N5	0.2326 (3)	0.80599 (18)	0.93231 (19)	0.0427 (7)	
H5C	0.2959	0.8370	0.8951	0.051*	
H5D	0.1861	0.7630	0.8972	0.051*	
N6	0.2461 (3)	0.8549 (2)	1.1263 (2)	0.0489 (7)	
H6C	0.1687	0.8327	1.1598	0.059*	
H6D	0.3122	0.8826	1.1661	0.059*	
Zn1	0.36240 (4)	0.74282 (2)	1.04903 (2)	0.03245 (12)	
Zn2	1.16044 (4)	0.54507 (3)	0.77627 (3)	0.04177 (13)	
Cl1	1.24823 (14)	0.45660 (8)	0.89729 (7)	0.0752 (3)	
Cl2	1.35279 (12)	0.62417 (13)	0.70905 (7)	0.1022 (5)	
Cl3	0.99364 (9)	0.64689 (6)	0.83974 (6)	0.0446 (2)	
Cl4	1.02570 (12)	0.46530 (7)	0.66406 (7)	0.0646 (3)	

### Atomic displacement parameters (Å^2^)

**Table:**

	*U*^11^	*U*^22^	*U*^33^	*U*^12^	*U*^13^	*U*^23^
C1	0.113 (4)	0.051 (3)	0.066 (3)	−0.026 (3)	0.019 (3)	0.013 (2)
C2	0.101 (4)	0.064 (3)	0.048 (2)	−0.003 (2)	0.010 (2)	0.015 (2)
C3	0.040 (2)	0.064 (2)	0.048 (2)	0.0067 (18)	0.0081 (16)	−0.0030 (18)
C4	0.0315 (18)	0.062 (2)	0.061 (2)	−0.0012 (17)	−0.0004 (16)	0.0065 (19)
C5	0.038 (2)	0.065 (3)	0.081 (3)	0.0135 (19)	−0.0173 (19)	0.003 (2)
C6	0.047 (2)	0.047 (2)	0.082 (3)	0.0155 (18)	−0.002 (2)	−0.007 (2)
N1	0.057 (2)	0.058 (2)	0.0594 (19)	−0.0205 (16)	0.0080 (15)	−0.0083 (16)
N2	0.068 (2)	0.0403 (17)	0.0335 (14)	0.0060 (15)	−0.0022 (13)	−0.0020 (12)
N3	0.0433 (16)	0.0411 (16)	0.0414 (14)	0.0048 (13)	−0.0033 (12)	−0.0036 (12)
N4	0.0363 (15)	0.0412 (16)	0.0419 (14)	−0.0021 (12)	−0.0094 (11)	0.0002 (13)
N5	0.0381 (15)	0.0406 (16)	0.0489 (16)	−0.0051 (13)	−0.0142 (12)	0.0005 (13)
N6	0.0381 (16)	0.0548 (19)	0.0539 (17)	0.0043 (14)	0.0014 (13)	−0.0109 (15)
Zn1	0.0331 (2)	0.0308 (2)	0.03334 (19)	−0.00077 (15)	−0.00142 (14)	0.00084 (15)
Zn2	0.0372 (2)	0.0524 (3)	0.0353 (2)	0.01035 (18)	−0.00777 (16)	−0.00588 (18)
Cl1	0.0995 (8)	0.0657 (7)	0.0594 (6)	0.0405 (6)	−0.0250 (6)	0.0033 (5)
Cl2	0.0491 (7)	0.2003 (17)	0.0572 (6)	−0.0341 (8)	0.0002 (5)	0.0172 (8)
Cl3	0.0429 (5)	0.0360 (5)	0.0546 (5)	0.0064 (4)	−0.0047 (4)	−0.0084 (4)
Cl4	0.0747 (7)	0.0603 (6)	0.0580 (6)	0.0128 (5)	−0.0195 (5)	−0.0288 (5)

### Geometric parameters (Å, º)

**Table:**

C1—C2	1.467 (5)	N1—H1C	0.8900
C1—N1	1.490 (5)	N1—H1D	0.8900
C1—H1A	0.9700	N2—Zn1	2.173 (3)
C1—H1B	0.9700	N2—H2C	0.8900
C2—N2	1.458 (5)	N2—H2D	0.8900
C2—H2A	0.9700	N3—Zn1	2.196 (2)
C2—H2B	0.9700	N3—H3C	0.8900
C3—N3	1.473 (4)	N3—H3D	0.8900
C3—C4	1.483 (5)	N4—Zn1	2.208 (3)
C3—H3A	0.9700	N4—H4C	0.8900
C3—H3B	0.9700	N4—H4D	0.8900
C4—N4	1.478 (4)	N5—Zn1	2.175 (2)
C4—H4A	0.9700	N5—H5C	0.8900
C4—H4B	0.9700	N5—H5D	0.8900
C5—N5	1.468 (4)	N6—Zn1	2.219 (3)
C5—C6	1.502 (5)	N6—H6C	0.8900
C5—H5A	0.9700	N6—H6D	0.8900
C5—H5B	0.9700	Zn2—Cl1	2.2546 (10)
C6—N6	1.468 (4)	Zn2—Cl2	2.2552 (12)
C6—H6A	0.9700	Zn2—Cl4	2.2654 (9)
C6—H6B	0.9700	Zn2—Cl3	2.2718 (9)
N1—Zn1	2.208 (3)		
			
C2—C1—N1	110.6 (3)	H2C—N2—H2D	108.2
C2—C1—H1A	109.5	C3—N3—Zn1	107.77 (19)
N1—C1—H1A	109.5	C3—N3—H3C	110.2
C2—C1—H1B	109.5	Zn1—N3—H3C	110.2
N1—C1—H1B	109.5	C3—N3—H3D	110.2
H1A—C1—H1B	108.1	Zn1—N3—H3D	110.2
N2—C2—C1	110.2 (3)	H3C—N3—H3D	108.5
N2—C2—H2A	109.6	C4—N4—Zn1	108.9 (2)
C1—C2—H2A	109.6	C4—N4—H4C	109.9
N2—C2—H2B	109.6	Zn1—N4—H4C	109.9
C1—C2—H2B	109.6	C4—N4—H4D	109.9
H2A—C2—H2B	108.1	Zn1—N4—H4D	109.9
N3—C3—C4	109.5 (3)	H4C—N4—H4D	108.3
N3—C3—H3A	109.8	C5—N5—Zn1	108.9 (2)
C4—C3—H3A	109.8	C5—N5—H5C	109.9
N3—C3—H3B	109.8	Zn1—N5—H5C	109.9
C4—C3—H3B	109.8	C5—N5—H5D	109.9
H3A—C3—H3B	108.2	Zn1—N5—H5D	109.9
N4—C4—C3	110.6 (3)	H5C—N5—H5D	108.3
N4—C4—H4A	109.5	C6—N6—Zn1	107.1 (2)
C3—C4—H4A	109.5	C6—N6—H6C	110.3
N4—C4—H4B	109.5	Zn1—N6—H6C	110.3
C3—C4—H4B	109.5	C6—N6—H6D	110.3
H4A—C4—H4B	108.1	Zn1—N6—H6D	110.3
N5—C5—C6	109.4 (3)	H6C—N6—H6D	108.6
N5—C5—H5A	109.8	N2—Zn1—N5	171.16 (11)
C6—C5—H5A	109.8	N2—Zn1—N3	95.43 (10)
N5—C5—H5B	109.8	N5—Zn1—N3	91.72 (10)
C6—C5—H5B	109.8	N2—Zn1—N1	79.79 (12)
H5A—C5—H5B	108.2	N5—Zn1—N1	94.79 (11)
N6—C6—C5	109.5 (3)	N3—Zn1—N1	92.04 (11)
N6—C6—H6A	109.8	N2—Zn1—N4	92.64 (11)
C5—C6—H6A	109.8	N5—Zn1—N4	93.80 (10)
N6—C6—H6B	109.8	N3—Zn1—N4	79.26 (10)
C5—C6—H6B	109.8	N1—Zn1—N4	167.95 (11)
H6A—C6—H6B	108.2	N2—Zn1—N6	94.08 (11)
C1—N1—Zn1	105.6 (2)	N5—Zn1—N6	79.69 (10)
C1—N1—H1C	110.6	N3—Zn1—N6	167.09 (10)
Zn1—N1—H1C	110.6	N1—Zn1—N6	98.21 (11)
C1—N1—H1D	110.6	N4—Zn1—N6	91.62 (10)
Zn1—N1—H1D	110.6	Cl1—Zn2—Cl2	111.51 (5)
H1C—N1—H1D	108.8	Cl1—Zn2—Cl4	113.03 (4)
C2—N2—Zn1	109.9 (2)	Cl2—Zn2—Cl4	110.45 (4)
C2—N2—H2C	109.7	Cl1—Zn2—Cl3	106.75 (4)
Zn1—N2—H2C	109.7	Cl2—Zn2—Cl3	108.28 (5)
C2—N2—H2D	109.7	Cl4—Zn2—Cl3	106.52 (4)
Zn1—N2—H2D	109.7		
			
N1—C1—C2—N2	−55.9 (5)	C4—C3—N3—Zn1	45.2 (3)
N3—C3—C4—N4	−55.4 (4)	C3—C4—N4—Zn1	35.9 (3)
N5—C5—C6—N6	−57.5 (4)	C6—C5—N5—Zn1	41.1 (4)
C2—C1—N1—Zn1	44.6 (4)	C5—C6—N6—Zn1	42.6 (3)
C1—C2—N2—Zn1	36.6 (4)		

### Hydrogen-bond geometry (Å, º)

**Table:**

*D*—H···*A*	*D*—H	H···*A*	*D*···*A*	*D*—H···*A*
N1—H1*D*···Cl1^i^	0.89	2.88	3.617 (3)	142
N1—H1*D*···Cl3^i^	0.89	2.95	3.677 (3)	140
N2—H2*C*···Cl1^ii^	0.89	2.56	3.426 (3)	165
N2—H2*D*···Cl3^iii^	0.89	2.51	3.395 (3)	171
N3—H3*C*···Cl1^ii^	0.89	2.67	3.500 (3)	155
N3—H3*D*···Cl1^i^	0.89	2.99	3.670 (3)	135
N3—H3*D*···Cl2^i^	0.89	2.85	3.600 (3)	143
N4—H4*C*···Cl4^iv^	0.89	2.63	3.437 (3)	152
N4—H4*D*···Cl2^iii^	0.89	2.76	3.581 (3)	153
N5—H5*C*···Cl4^iv^	0.89	2.58	3.431 (3)	160
N5—H5*D*···Cl3^i^	0.89	2.50	3.360 (3)	162
N6—H6*C*···Cl2^v^	0.89	2.91	3.646 (3)	141
N6—H6*D*···Cl3^iii^	0.89	2.91	3.652 (3)	142
N6—H6*D*···Cl4^iii^	0.89	2.90	3.609 (3)	138
C3—H3*B*···Cl3	0.97	2.82	3.662 (3)	146

Symmetry codes: (i) *x*−1, *y*, *z*; (ii) −*x*+2, −*y*+1, −*z*+2; (iii) *x*−1/2, −*y*+3/2, *z*+1/2; (iv) −*x*+3/2, *y*+1/2, −*z*+3/2; (v) *x*−3/2, −*y*+3/2, *z*+1/2.
